# Improving Service User Satisfaction of the Therapeutic Activities Provided in a Psychiatry Intensive Care Unit

**DOI:** 10.1192/bjo.2025.10458

**Published:** 2025-06-20

**Authors:** Sissira Valaparambil Sivasankaran, Shakir Wani, Matthew Day

**Affiliations:** Norfolk and Suffolk NHS Foundation Trust, Norwich, United Kingdom

## Abstract

**Aims:** Psychiatry intensive care units (PICU) are therapeutic environments that care for people with the most severe mental health conditions often characterized by heightened agitation, aggression, or self-harm behaviours. It is also one of the most restrictive units. Royal College of Psychiatrists standards for psychiatric intensive care units has given emphasis on the provision of therapeutic activities. We identified the scope to improve the choice, availability and accessibility of activities provided in PICU.

**Aim:** of the quality improvement project is to improve the service user satisfaction of therapeutic activities provided in the PICU from 60% to at least 80% in a duration of 6 months.

**Methods:** The principles of Quality improvement were followed. Did a process mapping, change ideas was collected from the whole team including service users, a driver diagram was created, changes were introduced, and impact was measured through Plan Do Study Act (PDSA) cycles.

Data was collected from service users on a scale of 1 to 5 on a daily basis in community meetings. Balance and process measures data was extracted from the records. The data was plotted as a run chart.

**Results:** The primary outcome measure used was service user satisfaction. Balance measures used included risk incidents (absconsion, physical and non physical assaults, self harm and sexual offences). The process measures included occupied bed days and volume of admissions or transfers.

Service user satisfaction score improved from 3 at base line to 4.2 out of 5, soon after the launch of the project. Median of rate of risk incidents reduced from 12 to 4. There was no difference in the process measures over the project period.

Change ideas tested included improving the communication between service users and staff, improving the communication between multidisciplinary staff members, improving the interests and enthusiasm of team, maintaining the enthusiasm in the therapeutic activities for both staff and service users, improving the understanding among staff of the therapeutic activities of interest in the service users and ensuring to maintain the material resources.

**Conclusion:** The project fostered a multidisciplinary approach, involving collaboration among psychiatrists, nurses, social workers, occupational therapists, service users and other allied health professionals. There was an inertia to initiate therapeutic activities in the ward, the project has helped to break this and the stereotypies about who needs to take initiatives on providing therapeutic activities.

